# Management and follow-up of gallbladder polyps: Joint guidelines between the European Society of Gastrointestinal and Abdominal Radiology (ESGAR), European Association for Endoscopic Surgery and other Interventional Techniques (EAES), International Society of Digestive Surgery – European Federation (EFISDS) and European Society of Gastrointestinal Endoscopy (ESGE)

**DOI:** 10.1007/s00330-017-4742-y

**Published:** 2017-02-09

**Authors:** Rebecca Wiles, Ruedi F. Thoeni, Sorin Traian Barbu, Yogesh K. Vashist, Søren Rafael Rafaelsen, Catherine Dewhurst, Marianna Arvanitakis, Max Lahaye, Marek Soltes, Julie Perinel, Stuart Ashley Roberts

**Affiliations:** 1 Department of Radiology, Royal Liverpool and Broadgreen University Hospitals NHS Trust, Liverpool, L78XP UK; 2 Department of Radiology and Biomedical Imaging, University of California, San Francisco, Medical School, San Francisco, CA USA; 3 4th Surgery Department, University of Medicine and Pharmacy “Iuliu Hatieganu”, Cluj-Napoca, Romania; 4 Section for Visceral Surgery, Department of Surgery, Kantonsspital Aarau, Aarau, Switzerland; 5 Department of General, Visceral and Thoracic Surgery, University Medical Center Hamburg-Eppendorf, Martinistrasse 52, 20246 Hamburg, Germany; 6 Department of Radiology, Clinical Cancer Centre, Vejle Hospital, University of Southern Denmark, Odense M, Denmark; 7 Department of Radiology, Mercy University Hospital, Grenville Place, Cork, Ireland; 8 Department of Gastroenterology, Erasme University Hospital ULB, Brussels, Belgium; 9 Department of Radiology, The Netherlands Cancer Institute, Amsterdam, The Netherlands; 10 1st Department of Surgery LF UPJS a UNLP, Kosice, Slovakia; 11 Department of Hepatobiliary and Pancreatic Surgery, Edouard Herriot Hospital, Lyon, France; 12 Department of Radiology, University Hospital of Wales, Cardiff, UK

**Keywords:** Gallbladder, Polyps, Neoplasms, Ultrasonography, Cholecystectomy

## Abstract

**Objectives:**

The management of incidentally detected gallbladder polyps on radiological examinations is contentious. The incidental radiological finding of a gallbladder polyp can therefore be problematic for the radiologist and the clinician who referred the patient for the radiological examination. To address this a joint guideline was created by the European Society of Gastrointestinal and Abdominal Radiology (ESGAR), European Association for Endoscopic Surgery and other Interventional Techniques (EAES), International Society of Digestive Surgery – European Federation (EFISDS) and European Society of Gastrointestinal Endoscopy (ESGE).

**Methods:**

A targeted literature search was performed and consensus guidelines were created using a series of Delphi questionnaires and a seven-point Likert scale.

**Results:**

A total of three Delphi rounds were performed. Consensus regarding which patients should have cholecystectomy, which patients should have ultrasound follow-up and the nature and duration of that follow-up was established. The full recommendations as well as a summary algorithm are provided.

**Conclusions:**

These expert consensus recommendations can be used as guidance when a gallbladder polyp is encountered in clinical practice.

***Key Points*:**

• *Management of gallbladder polyps is contentious*

• *Cholecystectomy is recommended for gallbladder polyps >10 mm*

• *Management of polyps <10 mm depends on patient and polyp characteristics*

• *Further research is required to determine optimal management of gallbladder polyps*

## Introduction

Gallbladder polyps are elevations of the gallbladder wall that project into the lumen. They are commonly detected on ultrasound scans of the abdomen, with a prevalence estimated between 0.3 and 9.5%. They may also be found following analysis of the gallbladder specimen following cholecystectomy [1–5].

Gallbladder polyps can be divided into pseudopolyps and true gallbladder polyps. Pseudopolyps are more common than true polyps. In a recent systematic review by Elmasry et al. 70% of suspected gallbladder polyps were pseudopolyps [6]. Pseudopolyps are most commonly cholesterol pseudopolyps but also include focal adenomyomatosis and inflammatory pseudopolyps. Pseudopolyps do not in themselves have malignant potential. True gallbladder polyps can be benign or malignant. Benign polyps are most commonly adenomas while malignant polyps are usually adenocarcinomas. There are rare types of benign and malignant true gallbladder polyps, including mesenchymal tumours, lymphoma and metastases [7]. Unlike the adenoma–carcinoma sequence that is well described for colonic polyps, the adenoma–carcinoma sequence in the gallbladder is less well understood. The evidence that exists, however, suggests that at least some gallbladder adenocarcinomas have arisen in pre-existing adenomas [8–10] and as such the adenoma–carcinoma sequence is likely, at least for some cases.

For the purposes of this review the term “gallbladder polyps” will be used, although the authors acknowledge that many apparent gallbladder polyps demonstrated on ultrasound scans will in fact be pseudopolyps.

Gallbladder cancer, most commonly adenocarcinoma, is a relatively rare form of cancer. The incidence varies significantly between different ethnic groups. High risk groups, e.g. Northern Indians and Native South Americans, see incidences of up to 27/100,000 while lower risk groups, such as Caucasian North Americans, have an incidence of 1.5/100,000 [11]. Gallbladder cancer carries a poor prognosis once it becomes advanced with 5-year survival less than 25% if tumour perforates the serosa (T3) or regional lymph nodes are involved (N1) (stage III) [12]. If the cancer is confined to the muscularis mucosa (stage I) or perimuscular connective tissue (stage II) the 5-year survival rates are much more favourable at 100% and 57–72%, respectively. It is important, therefore, that gallbladder cancer is detected and managed early. As gallbladder polyps are common but gallbladder cancer is rare, it is a diagnostic challenge to determine which polyps are likely to be malignant or undergo malignant transformation in order to determine which patients require cholecystectomy.

Several groups of authors have suggested follow-up guidelines for gallbladder polyps based on systematic review of the available literature [6, 7, 13–17]. As a result of factors such as lack of randomised controlled trials, mostly low quality evidence and inhomogeneity of studies, these follow-up guidelines differ. It may be difficult, therefore, for the practising radiologist, clinician or sonographer to know what to recommend when they encounter a gallbladder polyp. This was also suggested by the results from a survey of surgeons by Marangoni et al. [18] who found that there is inhomogeneity of surgical practice in the management of gallbladder polyps.

To address this the European Society of Gastrointestinal and Abdominal Radiology (ESGAR) sought to develop evidence-based consensus guidelines with the aim of answering several questions: which patients require cholecystectomy, which patients require ultrasound follow-up and what the frequency and duration of follow-up should be. A group consisting of gastrointestinal radiologists was formed for this purpose. As this is an issue that affects multiple medical disciplines—most commonly gastrointestinal surgeons and gastroenterologists—representatives from these disciplines were also included in compiling these guidelines. These guidelines are applicable to all patients in whom a gallbladder polyp is found on a conventional ultrasound scan.

## Methodology

In March 2015, ESGAR members were contacted via email for expressions of interest in contributing to these guidelines. The ESGAR Guidelines Committee appointed a chair (SAR) to oversee the guideline development, and the chair selected five of the respondents, on the basis of their experience in authorship of relevant literature or previous guideline development (RW, RT, SR, CD and ML). By consensus a second chair was then appointed to facilitate guideline development (RW). Through United European Gastroenterology, representatives from the International Society of Digestive Surgery (YV, SB and JP), the European Association for Endoscopic Surgery and other interventional techniques (MS) and the European Society of Gastrointestinal Endoscopy (MA) were invited to join the panel.

During the development process the principles of the AGREE II instrument were used wherever possible [19].

A literature search was performed to include potentially relevant articles published between January 1995 and October 2015. A summary of the search strategy can be found in Appendix. Abstracts of these articles were then evaluated and from these, a list of relevant articles compiled and sent to the group. Any additional suitable articles that were published between October 2015 and September 2016 found by the group could also be used as evidence. The literature primarily deals with gallbladder polyps demonstrated on conventional transabdominal ultrasound but other imaging modalities were also considered (recommendations and statements related to these can be found in the sections below).

Consensus was sought via a series of Delphi questionnaires, which were initially devised by the two chairs and approved by the group. The group were asked to score agreement with statements using a seven-point Likert scale (1 = strongly disagree, 7 = strongly agree) [20]. Agreement was determined if 75% of the group scored within 2 points; 75% agreement with a score of 6 or 7 meant that the recommendation was accepted; 75% agreement with scores of 1 or 2 meant the recommendation was rejected. Where consensus was not reached, a further Delphi round was performed. In total three Delphi rounds were required. The group were asked to grade the level of evidence using the GRADE system [21].

A draft manuscript was sent to the group by the coordinating chairs. The results were also discussed by group members at a panel meeting during the annual ESGAR meeting, Prague, 16 June 2016.

The final manuscript was reviewed by the ESGAR Guidelines Committee for approval prior to submission for publication.

## Recommendations and statements

**Fig. 1:**
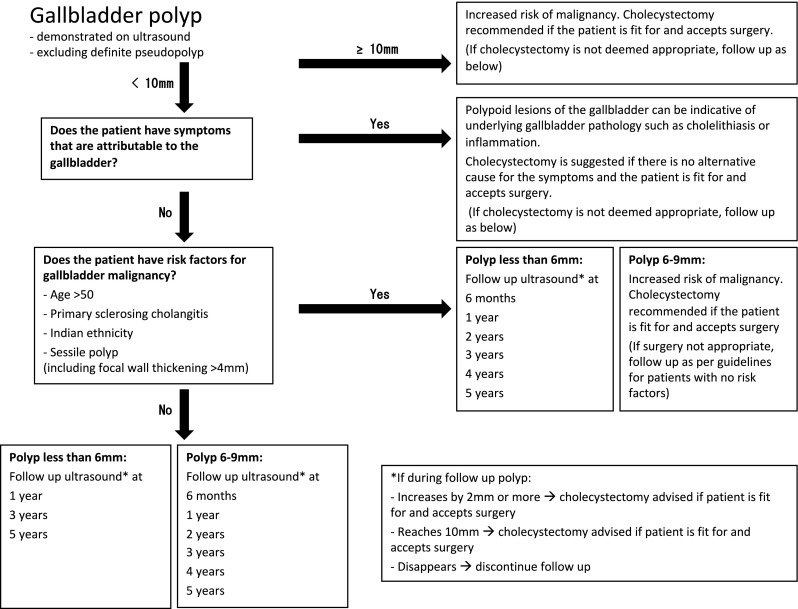
Management algorithm

A summary of the recommendations is described in the algorithm (Fig. 1). Each point in the algorithm will be discussed separately below. Diagnosis and follow-up are based on transabdominal ultrasound. In cases of multiple polyps the largest polyp should be used in deciding management. Grades of evidence as per the GRADE system [21] and agreement level of the group (in per cent) are included in brackets after each statement.

### Polyp definition

On ultrasound a gallbladder polyp is seen as an elevation of the gallbladder wall that protrudes into the lumen. It should not be mobile or demonstrate posterior acoustic shadowing (which would suggest it is more likely a calculus). It may be sessile or pedunculated.

A clearly infiltrating or large mass should be treated as a gallbladder cancer rather than a polyp.

**Fig. 2:**
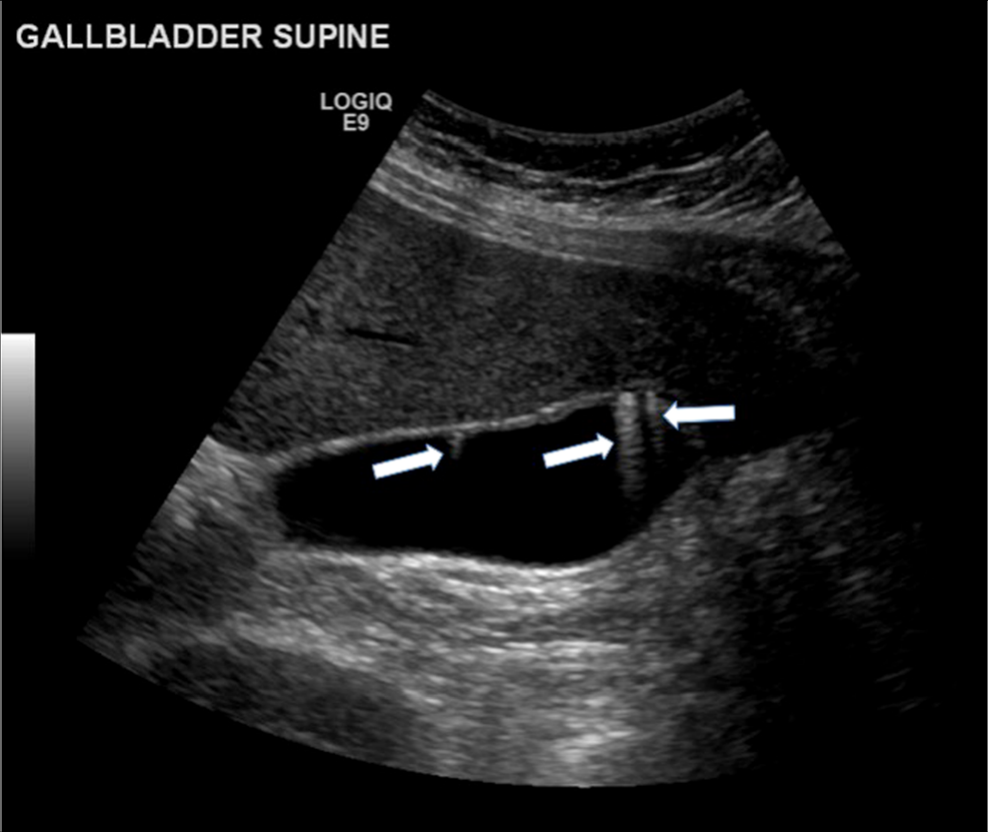
Pseudopolyps. This selected image from a transabdominal ultrasound scan demonstrates three separate pseudopolyps. Note the reverberation or “comet-tail” artefact posterior to the lesions (*arrows*)

If there is clear reverberation or “comet tail” artefact present posterior to the lesion (Fig. 2) this should be identified as a pseudopolyp (focal adenomyomatosis or a cholesterol polyp [22, 23]). The follow-up guidelines, therefore, do not need to be followed for these patients. Of note, not all pseudopolyps will demonstrate these findings.

### Polypoid lesion of the gallbladder greater than or equal to 10 mm—cholecystectomy is recommended if the patient is fit for and accepts surgery (moderate quality evidence, 89% agreement)

The evidence of 10 mm as a cut-off value for cholecystectomy is of moderate quality at best. This is due to a combination of lack of randomised controlled trial data, mainly retrospective studies and, in most cases, an arbitrary cut-off of 10 mm. In addition, because of the relatively low prevalence of gallbladder cancer, power calculation (an indication of the minimum number of subjects that need to be enrolled in a study to have sufficient statistical power to determine risk) are likely to produce very large numbers of patients, which has thus far not been achieved in any of the available literature. Despite this limited evidence level, it seems that there is reasonable consensus on the suggested recommendation in the published literature, based principally on the observation of a greater incidence of gallbladder carcinoma in the larger polyps [1, 7, 13–17, 24–36]. Moreover, there is evidence that suggests pseudopolyps tend to be smaller than true polyps. Thus, if cholecystectomy is performed for a gallbladder polyp 10 mm or larger then it is not only more likely that the lesion is malignant but if it is not malignant then it is more likely to be an adenoma and thus have malignant potential.

Some authors, such as Bhatt et al., have suggested higher threshold values for cholecystectomy [14]. The group concluded, however, that more evidence is available for the 10-mm threshold.

The group recognises that cholecystectomy may not be appropriate in all patients, e.g. patients with multiple comorbidities. In these patients discussion at a multidisciplinary team (MDT) meeting is suggested. Follow-up ultrasound may be useful in specific circumstances (e.g. if the patient is adamant that they do not want surgery unless the gallbladder polyp increases in size) but inappropriate in others (e.g. if patient comorbidity means that the patient would never be suitable for active treatment).

### Polypoid lesion of the gallbladder with patient’s symptoms attributable to the gallbladder—cholecystectomy is suggested if there is no alternative cause for the patient’s symptoms and the patient is fit for and accepts surgery (low quality evidence, 89% agreement)

It is unlikely that small gallbladder polyps themselves cause patient’s symptoms. There is evidence, however, that gallbladder polyps may be indicative of underlying inflammation or stone disease that may not have been detected on ultrasound [30, 37].

The relationship between symptoms and risk of malignancy is not established. Some authors, e.g. Kwon et al., suggest that if a patient with a gallbladder polyp has symptoms they are more likely to have a malignant polyp [29]. Park et al., however, found no relationship on multivariate analysis [1].

One small study by Jones-Monahan et al. demonstrated that of 45 symptomatic patients with gallbladder polyps and no calculi who underwent cholecystectomy, 93% experienced relief of symptoms [38].

French et al. concluded that, because the sensitivity and specificity of ultrasound for predicting histology in gallbladder polyps are low, patients with symptoms attributable to the gallbladder should have cholecystectomy, rather than follow-up ultrasound, when a polyp is present [39].

Overall the evidence level for most of the studies is low. The group concluded, however, that cholecystectomy is reasonable if there are no other explanations for the patient’s pain.

The group recognises that not all patients will be considered suitable for cholecystectomy for a variety of reasons. This includes patient comorbidity, patient preference or symptoms that are not convincingly attributable to the gallbladder. In these patients, the group recommends that patients be followed up as per the algorithm outlined above (unless for whatever reason the patient would never be suitable for active treatment).

### If cholecystectomy is not indicated because of the above reasons, the patient’s risk factors for gallbladder malignancy should be established and these patients should follow a more intensive management plan (see below). These risk factors are:

- **Age >50**
- **History of primary sclerosing cholangitis (PSC)**
- **Indian ethnicity**
- **Sessile polyp (including focal gallbladder wall thickening >4 mm)**

**(Low-moderate quality evidence, 78% agreement).**

### Age

As with most other cancers, the risk of a gallbladder polyp being malignant increases with increasing patient age. The quality of evidence that patients who are older require more regular follow-up or cholecystectomy for smaller polyps is low, mainly as a result of retrospective studies, inhomogeneous data reporting and, in most cases, arbitrarily selected cut-off values for age. The age threshold is variable across studies. Some studies use a threshold of 50 years [14, 15, 33, 40] whilst others suggest 57 [1], 60 [24, 29, 36] or 65 years [41]. Currently there is insufficient data to determine what the most appropriate threshold is, or indeed whether patients of different ages should be treated differently. Nevertheless the group felt that, given the fact that gallbladder cancer is definitely more common in older patients, age should be established as a risk factor in the guidelines. The group concluded to use 50 as the threshold for this but recognises that this is based on consensus rather than conclusive scientific evidence.

### Primary sclerosing cholangitis (PSC)

The American Association for the Study of Liver Diseases and the European Association for the Study of the Liver both recommend cholecystectomy for patients with PSC and a gallbladder polyp, irrespective of size [42, 43]. This is due to an increased risk of malignancy in gallbladder polyps in patients with PSC. A study by Said et al. demonstrated that in 18 patients with gallbladder masses and PSC, malignancy was present in 56% of polyps in cholecystectomy specimens [44], although these masses ranged in size from 5 to 35 mm. Similarly in another study by Buckles et al. 57% of 14 gallbladder masses were malignant and 33% of the benign adenomas demonstrated dysplasia [45], but again these lesions ranged in size from 6 to 30 mm.

Other studies, however, have demonstrated fewer malignant gallbladder polyps in patients with PSC. A more recent study of patients with PSC by Eaton et al. demonstrated that only 14% of 14 gallbladder polyps were malignant [46].

The quality of evidence in this area is low because of low sample sizes, mostly retrospective studies and lack of primary outcome data. Despite this, however, the evidence that is available suggests that in patients with PSC a gallbladder polyp is more likely to be malignant.

There is, however, an increased risk of complications in patients with PSC undergoing cholecystectomy, particularly those with advanced cirrhosis [46].

For the above reasons, the group felt that there was insufficient data to support cholecystectomy in all patients with PSC and a gallbladder polyp, because of the potential increased morbidity. The group felt, therefore, that these patients should undergo a more intensive follow-up and have a lower threshold for cholecystectomy than non-PSC patients. However, the group recognises that cholecystectomy may not be appropriate in all patients because of patient comorbidities. As such, if cholecystectomy cannot be performed safely the follow-up schedule outlined in the algorithm could be adopted.

### Indian ethnicity

A large study by Aldouri et al. involving 2359 patients with gallbladder polyps demonstrated that on multivariate analysis patients of Indian ethnicity had a significantly higher prevalence of gallbladder cancer: 5.5% versus 0.08% [24]. Whilst this is the only paper, to our knowledge, that has specifically studied this risk factor, given that it is a relatively large cohort and of a moderate level of evidence, the group felt that it was reasonable to include this in the guidelines. More studies, preferably prospective, would be required to confirm the recommendations.

### Sessile polyp (including focal GB wall thickening >4 mm)

A recently published systematic review by Bhatt et al. demonstrated that sessile morphology in a gallbladder polyp is an independent risk factor for malignancy, increasing the risk by a factor of 7.32 (95% confidence interval 4.18–12.82) [14]. This was similar to a finding in a retrospective study by Kwon et al. of 291 patients with a gallbladder polyp on cholecystectomy that demonstrated an odds ratio of 7.70 (95% confidence interval 2.48–23.95) [29].

A larger retrospective study by Park et al. of 689 patients with gallbladder polyps [1] demonstrated that sessile morphology was a risk factor on univariate but not multivariate analysis. It should be noted that the majority of patients in this study were followed up with ultrasound rather than cholecystectomy.

Overall the group felt that sessile morphology should be included as a significant risk factor for malignancy in the guidelines.

It has been suggested by some authors that gallbladder wall thickening is an indication of malignancy. Two studies have demonstrated increased risk of malignancy in patients with wall thickening. Aldouri et al. [24] demonstrated that wall thickening >5 mm and wall irregularity were both independent risk factors for malignancy on multivariate analysis (although these were secondary outcomes in the study). This subgroup of patients, however, did not have gallbladder polyps.

Another study by Zhu et al. [47] of 29 elderly patients in whom gallbladder cancer was found incidentally on cholecystectomy showed that wall thickening >4 mm was an independent variable for gallbladder cancer (*P* = 0.002). This study, however, did not deal only with patients who had cancer in polyps. It is also limited by its small sample size and patient demographics.

A further study of 361 patients by Choi et al. [27] demonstrated that patients with gallbladder wall thickening (defined as 3 mm or more) were more likely to undergo subsequent cholecystectomy for whatever reason, compared to patients without thickening. In this study, however, no patients subsequently developed malignancy and as such the relevance of this study in determining that wall thickening is associated with malignancy is doubtful. It may indicate, however, that patients with wall thickening are more likely to develop symptoms leading to cholecystectomy, but this is speculative.

Again there are no prospective studies or randomised controlled trials. As such the level of evidence is low and further studies are needed.

The group feels that patients with focal wall thickening >4 mm should be treated the same as patients with sessile polyps.

### Other risk factors

The group recognises that other factors probably make gallbladder malignancy more likely. Some studies suggest that solitary gallbladder polyps, for example, are more likely to be malignant than multiple polyps [6, 14], although the increased risk of malignancy in the systematic review by Bhatt et al. was only 2.05. Other studies, however, have shown this not to be significant on multivariate analysis [24]. There are no robust data, to our knowledge, that suggest asymptomatic multiple polyps are less likely to be malignant than asymptomatic solitary polyps. The group concluded that a solitary polyp should not be included as a specific risk factor.

People of Indian ethnicity, as described above, appear to have increased risk of malignancy in gallbladder polyps [24]. Other ethnic groups, for example East Asians, also appear to be at high risk of gallbladder cancer [13]. To the best of our knowledge, however, there are no studies that have directly compared the prevalence of malignancy in gallbladder polyps in ethnic groups other than Indian. As such, whilst it may be that polyps in other ethnic groups are more likely to be malignant, this has not been fully established and as such was not included in the guidelines. This is again an area for future research.

Some authors have suggested that the presence of gallstones may be a risk factor for malignancy in gallbladder polyps. Aldouri et al. [24], for example, demonstrated that the presence of gallstones was an independent risk factor but with borderline significance. Park et al., however, found that gallstones were not an independent risk factor on multivariate analysis [1]. Again the evidence level in this area is low. The group concluded that there was insufficient evidence to include gallstones as a strong risk factor in the guidelines, but note that some of these patients are likely to be symptomatic and as such will undergo cholecystectomy anyway.

### If the patient has risk factors for gallbladder malignancy and a polyp 6–9 mm, cholecystectomy is recommended if the patient is fit for and accepts surgery (low–moderate quality evidence, 78% agreement)

For the reasons stated above, patients with the above risk factors have a higher risk of gallbladder malignancy. As such the threshold of 10 mm as an indication for cholecystectomy should be lowered in these patients to 6 mm.

As for polyps 10 mm or greater, if the patient is not fit for surgery, either because of comorbidities or patient choice, the decision to perform follow-up will be based on the individual case in question. This may require MDT discussion. If a follow-up approach is decided upon, follow-up as per patients with no risk factors is advised.

### If the patient has either

**No risk factors for gallbladder malignancy and a gallbladder polyp of 6–9 mm or**

**Risk factors for malignancy and a gallbladder polyp 5 mm or less**

**Follow-up ultrasound of the gallbladder is recommended at 6 months, 1 year and then yearly up to 5 years.**

**If the patient has no risk factors for malignancy and a gallbladder polyp of 5 mm or less follow-up is advised at 1 year, 3 years and 5 years**

**(Low quality evidence, 78% agreement).**

Small gallbladder polyps are less likely to be malignant, or have malignant potential than larger polyps [48]. In a small number of cases, however, malignancy has been found in polyps <6 mm [24, 35]. Perhaps more importantly adenomas have more commonly been found in polyps <6 mm. Roa et al., for example, found that of 32 adenomas found following cholecystectomy 47% were <5 mm [49]. Another study by Kubota et al. [50] demonstrated that two out of the seven gallbladder polyps <5 mm found following cholecystectomy were adenomas.

Given the potential risk of malignancy, the group felt that an early follow-up ultrasound scan (at 6 months) followed by a scan at 12 months was warranted for patients with gallbladder polyps <6 mm and risk factors or for patients with larger polyps 6–9 mm and no risk factors. If a small gallbladder polyp were malignant at the time of the first scan then it is more likely to demonstrate growth by 6 months. The group did not feel this 6-month initial scan was required in patients with very low risk (i.e. no risk factors and polyp <6 mm).

The rest of the follow-up schedule suggested is at yearly intervals. This is to attempt to detect any small adenomas that undergo malignant change.

As described earlier, the adenoma–carcinoma sequence for gallbladder polyps seems likely, but there is insufficient data to suggest how long an adenoma is likely to be present before undergoing malignant change. In one study by Park et al. a polyp took 7 years to grow [32]. Wiles et al. demonstrated in a systematic review that gallbladder polyps that do grow appear to do so slowly [51]. The group concluded that a 5-year follow-up should be advised.

The group recognises that, again, there is a lack of robust data on which these recommendations have been made and that large prospective studies are needed to establish good evidence.

### If during follow-up gallbladder polyp increases by 2 mm or more cholecystectomy advised (moderate quality evidence 78% agreement)

Few gallbladder polyps grow on follow-up. A recent systematic review by Bhatt et al. [14] found that 93% of polyps did not increase in size. Growth rate, however, appears to be a potential risk factor for malignancy. Cairns et al. [25] demonstrated that in 467 patients with gallbladder polyps who underwent ultrasound follow-up, progression in size was predictive of malignancy or malignant potential. This study was limited, however, by a low prevalence of malignant or potentially malignant lesions (3.7%). In addition the progression in size was not defined.

Park et al. demonstrated that in 1558 patients with gallbladder polyps, 33 of which proved to be neoplastic, 25% of gallbladder polyps that increased in size were neoplastic [32].

A systematic review [51] by Wiles et al. that looked at growth of gallbladder polyps concluded that there were insufficient data to conclude what rate of growth in a gallbladder polyp is suggestive of malignancy. This was mainly due to growth rate not being reported in most studies, as well as small numbers of neoplastic polyps. In one study by Shin et al. growth rate of >0.6 mm/month was associated with malignancy on univariate but not multivariate analysis [34], although only 20 patients out of 145 had a neoplastic polyp.

In summary, it appears that increase in size may be a predictor for neoplasia, but no specific size increase has been established. The group felt that a 1-mm increase was too small because of differences in scanning techniques and ultrasound resolution but that 2 mm would more likely be representative of true growth. This is supported by evidence from a study by Sugiyama et al. [23] who demonstrated that in 58 patients who underwent conventional ultrasound and cholecystectomy for gallbladder polyps, the size of the polyp on ultrasound was within 2 mm of the size on cholecystectomy and was also within 2 mm of the size measured on EUS. Thus a size increase of 2 mm on ultrasound is likely to represent a true size increase, rather than being a spurious finding.

### If during follow-up gallbladder polyp reaches 10 mm cholecystectomy advised (moderate quality evidence, 100% agreement)

Gallbladder polyps 10 mm or greater are more likely malignant, as described above and as such cholecystectomy is advised.

### If during follow-up gallbladder polyp disappears discontinue follow-up (moderate quality evidence, 100% agreement)

If the gallbladder polyp disappears then it was likely a pseudopolyp and does not require further follow-up. This is assuming that there were no limitations to the quality of the scan (such as a non-distended gallbladder).

### In some cases, decision regarding cholecystectomy may be reached following multidisciplinary discussion

In some cases a patient may meet the criteria for cholecystectomy but the surgeon to whom the patient has been referred may not consider this the appropriate course of action. This may include patients with significant comorbidities, advanced age or due to patient choice. In these cases multidisciplinary discussion, including between the surgeon and radiologist, is advised.

### Primary investigation should be with abdominal ultrasound. Routine use of other imaging modalities is not recommended. In some centres with appropriate expertise and resources, alternative imaging modalities (such as endoscopic ultrasound) may be useful to aid decision-making in difficult cases (low quality evidence, 100% agreement)

Alternative imaging modalities have shown promising results in some studies on small numbers of patients.

EUS has been shown to be more accurate than conventional ultrasound in some small studies. In a study by Sugiyama et al. [23] of 58 patients with suspected gallbladder polyp who underwent cholecystectomy, EUS correctly distinguished between true and pseudopolyps in 97%. The figure for conventional ultrasound was 76% (which was statistically significant). In another study by Cheon et al. [52] of 94 patients with gallbladder polyps less than 20 mm who underwent cholecystectomy the diagnostic accuracy of EUS and conventional ultrasound was 80.9% and 63.9%, respectively (which was statistically significant), although for gallbladder polyps less than 11 mm these figures were 79.7% and 72.4% which may be more relevant to these guidelines.

Contrast enhanced ultrasound (CEUS) has been used to assess gallbladder polyps in some studies. In a multicentre study by Zheng et al. [53] of 116 patients with gallbladder polyps who underwent cholecystectomy it was demonstrated that CEUS increased diagnostic accuracy for characterisation of the lesions for gallbladder polyps >10 mm but not <10 mm. In a study by Liu et al. [54] of 83 patients with gallbladder polyps who underwent cholecystectomy and CEUS, whilst some of the imaging characteristics appeared to correlate with the histological diagnosis, these were not statistically significant.

Jang et al. [55] studied 144 patients who underwent cholecystectomy and high resolution ultrasound (HRUS). The high resolution technique involved using a high frequency probe and selective use of harmonic imaging. They found that for HRUS and EUS the sensitivity and specificity for diagnosing malignancy were similar (sensitivity 89.6% and 86.2%, specificity 86.9% and 86.9%, respectively). This study, however, did not compare HRUS with conventional ultrasound and did not assess accuracy at differentiating adenomas from pseudopolyps. Perhaps most importantly, the authors only studied gallbladder polyps >10 mm, which introduces potential bias and means the findings of the study cannot be applied to the management of patients with small polyps.

Some authors have studied the use of computed tomography (CT) in diagnosing gallbladder polyps. Furukawa et al. [56] demonstrated that all gallbladder polyps that were histologically confirmed following cholecystectomy were detected by contrast enhanced CT. The study was limited by a small sample size, however, with only five gallbladder polyps less than 11 mm in size. In a study by Lou et al. of 32 patients [57], CT biliary cystography was evaluated and the detection rates for gallbladder polyps were comparable with conventional ultrasound (detection rates of 93.8% (90/96) and 96.9%, respectively). This study mainly involved polyps less than 10 mm (86% of polyps). Although this is promising early data, the numbers were again small.

Magnetic resonance imaging (specifically diffusion weighted imaging, DWI) was studied by Irie et al. [58] who demonstrated that ADC values of benign gallbladder polyps were higher than malignant lesions. This evaluated only 23 patients and all polyps were 10 mm or larger. As such the results cannot be applied generally.

In summary, alternative imaging modalities, particularly EUS, may provide additional information in the diagnosis of gallbladder polyps. At present, however, there is insufficient data to suggest that they should be used ahead of conventional ultrasound in the investigation of gallbladder polyps. In addition transabdominal ultrasound is a relatively low cost, low risk and widely available technique which means that transabdominal ultrasound-based guidelines can be followed by most, if not all, European centres, rather than in specific specialist centres. Some centres with sufficient resources and expertise may find the additional information available useful, especially in patients for whom cholecystectomy may have additional risk.

## Limitations

### Quality of evidence

The level of evidence in most of the studies on which these guidelines are based is low or moderate at best as mentioned throughout this report. Most of the studies are retrospective and from single centres and many are biased, most commonly by the fact that patients who underwent cholecystectomy had large polyps or symptoms. In addition the rarity of gallbladder cancer means that studies with large sample sizes would be required to truly determine which gallbladder polyps are likely to undergo malignant change. The majority of studies only achieved small numbers. This makes formulating guidelines for the management of small polyps particularly difficult, which is reflected in the inhomogeneity of recommendations from the authors of systematic reviews [6, 7, 13–17]. This also explains why only two of the recommendations described above are based on moderate quality evidence and had 100% agreement between the authors—“*If during follow-up gallbladder polyp reaches 10 mm cholecystectomy advised”* and “*If during follow-up gallbladder polyp disappears discontinue follow-up”.*

A large, longitudinal multicentre trial is required to reliably answer the question of which patients require cholecystectomy, which patients require ultrasound follow-up and what the frequency and duration of that follow-up should be. The group wishes to stress that the development and publication of these guidelines should not preclude further research into this area.

### Cost of implementing the guidelines

The group proposes infrequent but long follow-up for gallbladder polyps. Estimating the cost of implementing these guidelines is difficult. Cairns et al. [25] studied 986 patients with gallbladder polyps and looked at the cost-effectiveness of ultrasound surveillance. They estimated that the surveillance programme would prevent 5.4 gallbladder cancers per 1000 patients scanned annually. On the basis of a number of pricing assumptions and 6-monthly ultrasound surveillance, they estimated that ultrasound surveillance was cost-effective when the cost of 5.4 gallbladder cancers per 1000 patients was taken into account. The authors made a number of assumptions in the study, including that all neoplastic polyps would become malignant. Whilst this data is not robust, it can be suggested that a surveillance programme may be cost-effective. Scanning at 12-monthly intervals may further increase this saving.

### Adherence to guidelines

The group aimed to make the guidelines applicable to hospitals throughout Europe. This included the suggestion of a purely conventional ultrasound-based follow-up programme. The guidelines have been formulated into an algorithm that, it is hoped, will be unambiguous and easy to follow.

### Patient involvement

To our knowledge there is no specific European patient focus group. The acceptance of these guidelines by patients is likely to vary across Europe. It was, therefore, not possible to include patient groups in the formulation of these guidelines as recommended in AGREE II. Conventional transabdominal ultrasound is, however, non-invasive, radiation- and contrast-free and relatively quick and simple to perform. We anticipate, therefore, that there should not be any specific barriers to patient acceptance of these guidelines, providing that the referring clinician describes to the patient the rationale for the scan and entry into a follow-up algorithm. This may also provide an opportunity for further research.

## Updating the guidelines

The group suggests that the guidelines are updated in September 2021 or earlier if robust new evidence becomes available that would significantly modify the recommendations.

## Appendix

**Table 1:** The literature search strategy is outlined below. MEDLINE and EMBASE databases were searched using search terms “gallbladder” and “polyp”, excluding case studies and limited to English language papers. Abstracts of all articles published between January 1995 and October 2015 were then reviewed.

1	Medline	(“gall bladder*” OR gallbladder*).ti	17699
2	Medline	polyp*.ti	60841
3	Medline	1 AND 2	363
4	Medline	3 NOT “case report*”	346
5	Medline	4 [Limit to: (Language English)]	250
6	EMBASE	4 [Limit to: (Language English)]	248
7	Medline, EMBASE	Duplicate filtered: [4 [Limit to: (Language English)]], [4 [Limit to: (Language English)]]	498 493 Unique results 5 Duplicate results

## Abbreviations

ADC: apparent diffusion coefficient
AGREE: Appraisal of Guidelines for Research & Evaluation
CEUS: contrast enhanced ultrasound
CT: computed tomography
DWI: diffusion weighted imaging
ESGAR: European Society of Gastrointestinal and Abdominal Radiology
EUS: endoscopic ultrasound
GRADE: Grading of Recommendations Assessment Development and Evaluation
HRUS: high resolution ultrasound
MDT: multidisciplinary team
PSC: primary sclerosing cholangitis
